# Airway protease availability governs a reversible shift between syncytial and cell-free spread

**DOI:** 10.1128/jvi.00727-26

**Published:** 2026-09-14

**Authors:** Joaquin Rodriguez Galvan, Maren de Vries, Shiraz Belblidia, Ashley Fisher, Meike Dittmann

**Affiliations:** 1 Microbiology Department, New York University Grossman School of Medicine 12296, New York, New York, USA; 2 Department of Medicine, New York University Grossman School of Medicine 12296, New York, New York, USA; University of Minnesota Twin Cities, Minneapolis, Minnesota, USA

**Keywords:** paramyxoviruses, TMPRSS, furin, syncytia, HPIV3, RSV, viral spread

## Abstract

Paramyxoviruses such as respiratory syncytial virus (RSV) and human parainfluenza virus 3 (HPIV3) spread in the airway by releasing virions and by forming syncytia. Spread phenotypes are typically attributed to the fusion protein cleavage site, yet it remains unclear whether, and if so, how, host protease context biases syncytial versus cell-free spread. We compared RSV and HPIV3—two closely related viruses with overlapping epithelial tropism but distinct F cleavage-site composition (polybasic, furin-type for RSV versus a distinct, less polybasic cleavage-site motif for HPIV3)—in respiratory cell lines and primary airway cells under defined *in vitro* protease conditions. In multi-cycle infections, furin-dominant, trypsin-like–low environments produced the largest syncytia, whereas trypsin-like–high environments favored smaller syncytia and more cell-free spread. Adding exogenous TMPRSS2 shifted HPIV3 toward cell-free spread with minimal impact on RSV. Using serine protease inhibitors (SERPINs) as protease-selective inhibitors, we show that inhibiting specific proteases restricts infection and remodels syncytium architecture, consistent with protease switching. Together, these results indicate that host protease availability is a reversible control point for respiratory virus dissemination. This framework has direct implications for protease-targeting therapeutics. Selective inhibition may reroute fusion activation and shift spread modality toward more syncytia-associated cytopathic effects rather than uniformly suppressing dissemination.

**IMPORTANCE:**

Respiratory syncytial virus and human parainfluenza virus 3 are leading causes of pneumonia and bronchiolitis in infants, the elderly, and immunocompromised patients, and disease severity tracks with how efficiently these viruses spread through the airway. Spread has long been attributed to a fixed feature of the viral fusion protein. Here, we show instead that the host airway environment—specifically, the proteases that activate viral fusion proteins—acts as a reversible switch between two modes of spread: virus release and cell-to-cell fusion. Because this switch can be moved, protease-targeting drugs may not simply block infection but redirect how the virus spreads, with consequences for tissue damage. Recognizing protease availability as a controllable determinant of dissemination reframes how we think about respiratory virus pathogenesis and offers a rationale for tuning, rather than only suppressing, viral spread with host-directed therapeutics.

## INTRODUCTION

Human respiratory paramyxo- and pneumoviridae are major causes of pediatric viral pneumonia and bronchiolitis worldwide (1, 2). These viruses primarily infect the respiratory epithelium and can cause disease ranging from mild upper respiratory tract (URT) infection to life-threatening lower respiratory tract (LRT) involvement in infants, the elderly, and the immunocompromised (3). Clinical severity is closely tied to the kinetics and efficiency of viral dissemination, including the ability to transition from the URT to the LRT and trigger inflammatory cascades (4, 5). Identifying the viral and host determinants that dictate the mode and magnitude of spread is therefore essential to understanding pathogenesis across these families of viruses.

Across paramyxoviruses, entry is mediated by an attachment glycoprotein and the fusion (F) glycoprotein (6–8). Attachment triggers F to undergo a large conformational rearrangement that drives membrane merger and delivery of the viral ribonucleoprotein into the cytoplasm (8). F is synthesized as an inactive precursor (F0) and must be cleaved into disulfide-linked F1 and F2 subunits to become fusion-competent (9). Cleavage occurs at a basic motif immediately upstream of the fusion peptide, which forms the new N-terminus of F1; proteolysis is therefore the step that liberates the fusion peptide for membrane insertion. Polybasic motifs matching the furin consensus (minimally R-X-X-R↓, optimally R-X-K/R-R↓) are processed intracellularly by furin, whereas monobasic or suboptimal motifs depend on trypsin-like serine proteases supplied by the host airway. Because this same activation step governs fusion at both the virion and infected-cell surface, it provides a mechanistic lever that can bias infection toward cell-free spread or toward syncytia-dependent cell–cell dissemination. Polybasic cleavage sites are classically associated with processing by furin and related proprotein convertases (PCSKs), but accumulating evidence indicates that activation is shaped by the broader airway proteolytic landscape (10–12). Accordingly, trypsin-like serine proteases (including TMPRSS family members), as well as host antiproteases, can contribute to—or restrict—fusion activation in a context-dependent manner (13).

Beyond entry, F expressed at the plasma membrane of infected cells can drive lateral cell–cell fusion to form multinucleated syncytia, providing a direct route of dissemination within the epithelium (6, 14–16). This strategy can reduce exposure to neutralizing antibodies but is also cytopathic and inflammatory (16). As a result, the extent to which infection proceeds via syncytia versus cell-free spread is likely to have important consequences for tissue damage and disease severity.

Because both entry and syncytium formation depend on proteolytic activation, the local airway protease repertoire is likely a key regulator of paramyxovirus fitness. However, how this environment varies along the respiratory tract—and how those differences map onto spread modality—remains poorly defined. This raises a fundamental question: does the viral F cleavage motif dictate a fixed spread strategy, or is spread a plastic trait shaped by the available host proteases?

In this study, we addressed this question by comparing human parainfluenza virus 3 (HPIV3) and respiratory syncytial virus (RSV), two clinically important respiratory paramyxoviruses that infect the airway epithelium (2, 17, 18). Although HPIV3 and RSV belong to separate families (Paramyxoviridae and Pneumoviridae, respectively) and their F proteins share only ~44% amino acid similarity (~20% identity), both F proteins adopt the conserved class I fusion architecture and require the same F0→F1 + F2 activation step. Despite this shared mechanism and overlapping epithelial tropism, RSV accounts for a substantially higher global disease burden and mortality rate than HPIV3 (2). We therefore leveraged HPIV3 and RSV as a comparative pair: two closely related respiratory viruses with overlapping epithelial tropism but sharply divergent disease burden. This design tests whether differences in dissemination reflect virus-intrinsic fusion machinery (e.g., cleavage-site–encoded constraints) or instead emerge from differences in how each virus engages the host airway protease environment. Using laboratory cell lines and primary human airway models, we found that differences in syncytium formation correlate with the relative abundance of furin versus trypsin-like serine proteases. By experimentally modulating this proteolytic environment through targeted enzyme supplementation or inhibition, we demonstrate that spread modality is a reversible switch. Together, these findings indicate that the host protease environment is a dominant determinant of viral dissemination and may help explain distinct pathological profiles of HPIV3 and RSV.

## RESULTS

### Host cell context shapes HPIV3 and RSV fusion phenotypes *in vitro*

**Fig 1:**
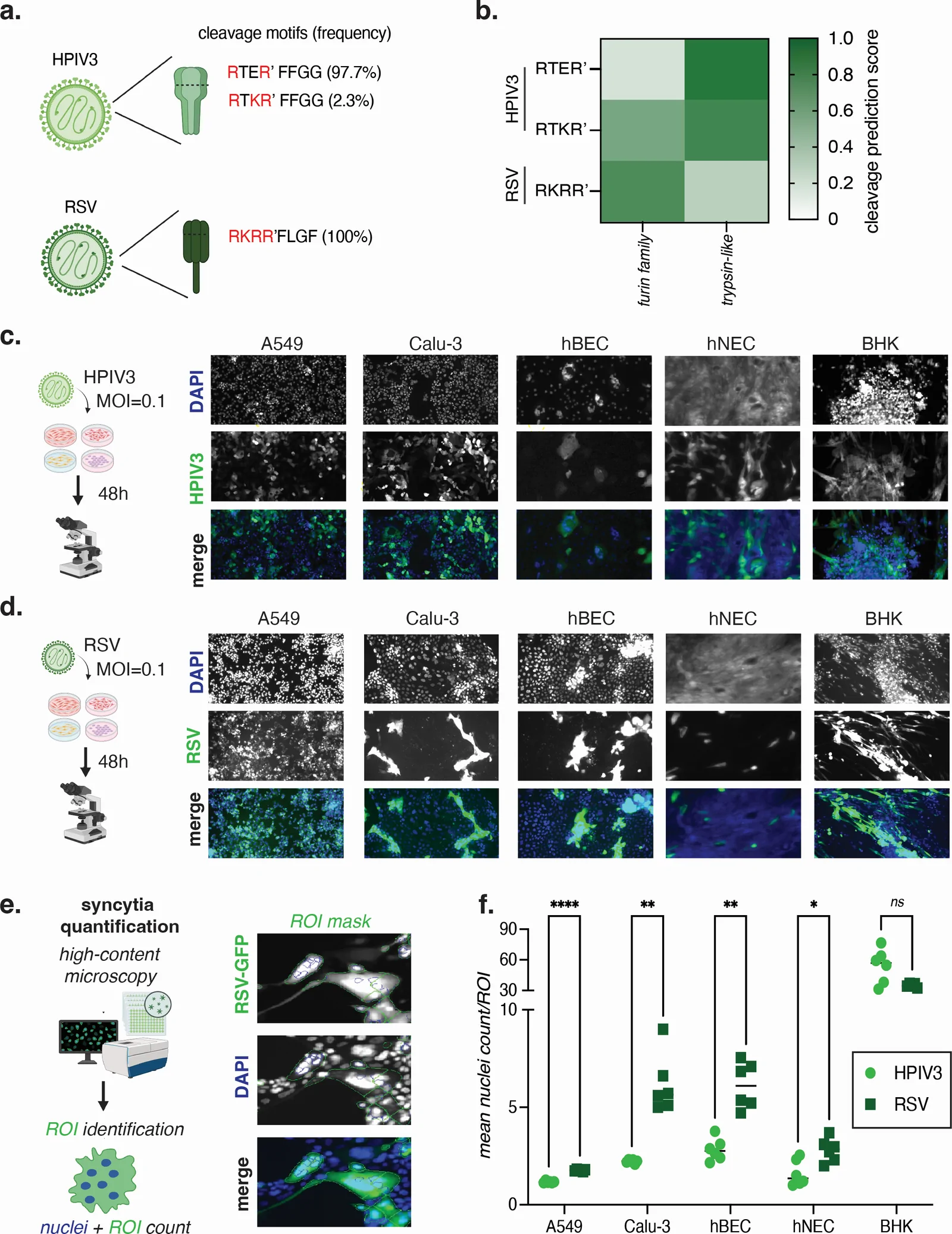
Experimental overview and quantification of syncytium formation in HPIV3- and RSV-infected cell lines and primary airway cells. (**a**) Schematic of HPIV3 and RSV type A fusion (F) protein cleavage sites; percentages indicate frequency among sequenced isolates (NCBI; HPIV3, 1,904 sequences; RSV, 14,664 sequences). (**b**) *In silico* cleavage predictions for HPIV3 and RSV F proteins generated using ProP 1.0 and PeptideCutter (default parameters). (**c**) Representative high-content fluorescence microscopy images of HPIV3-infected cells at 48 h post-infection (hpi) following infection at MOI 0.1 in A549, Calu-3, primary human bronchial epithelial cells (hBEC), human nasal epithelial cells (hNEC), and BHK cells. DAPI marks nuclei and GFP marks infected cells. *n* = 10 fields per condition. (**d**) Representative high-content fluorescence microscopy images of RSV-infected cells at 48 hpi following infection at MOI 0.1 in A549, Calu-3, hBEC, hNEC, and BHK cells. DAPI marks nuclei, and GFP marks infected cells. *n* = 10 fields per condition. (**e**) Workflow for high-content quantification of syncytium size. Regions of interest (ROIs) were defined by GFP signal and nuclei were identified by DAPI for calculation of nuclei per ROI. (**f**) Syncytium size quantified as nuclei per ROI across the indicated cell types. Data points represent field-level measurements (*n* = 10 fields per condition). Statistical significance was assessed by two-way ANOVA. * *P* < 0.05, ** *P* < 0.01, *** *P* < 0.001, **** *P* < 0.0001, ns = not significant.

To determine how host protease usage relates to viral spread, we contrasted HPIV3 and RSV, two closely related respiratory viruses with distinct predicted protease dependencies. We first asked whether differences in F protein cleavage site sequence could, on their own, account for differences in spread phenotype. HPIV3 F carries a single cleavage site with two circulating variants (RTER and RTKR), the RTER variant predominating among sequenced isolates (Fig. 1a). RTKR matches the optimal furin consensus, whereas the acidic residue at P2 in RTER is disfavored by furin, predicting greater dependence on trypsin-like proteases. RSV F, by contrast, is cleaved at two furin sites (RARR↓ and RKRR↓) that flank the p27 peptide and must both be processed for fusion competence (Fig. 1a). Consistent with these sequences, *in silico* prediction assigns both viruses’ cleavability by furin and trypsin-like proteases, with RSV showing a stronger furin bias (Fig. 1b).

We next tested how these predicted cleavage differences map onto *in vitro* spread phenotypes by performing multi-cycle infections across a panel of immortalized cell lines and primary human airway cells. We infected different cells at low multiplicity of infection (MOI) and measured viral spread by microscopy at 48 h post-infection (hpi) and quantified syncytium size using high-content imaging (infected regions defined by green fluorescent protein [GFP]; nuclei by DAPI) (Fig. 1c through f). The results revealed pronounced, cell type–dependent differences in fusion that were larger in magnitude than any differences suggested by cleavage-site sequence alone: HPIV3 produced minimal multinucleation in A549 cells and only small syncytia in Calu-3, primary human bronchial epithelial cells (hBEC), and human nasal epithelial cells (hNEC) (averages ~1.5 to 2.7 nuclei per infected region), whereas in BHK cells HPIV3 formed large multinucleated structures (average ~60 nuclei per structure) (Fig. 1f). RSV formed larger and more extensive syncytia than HPIV3 across most cell types (Fig. 1c, d and f), with BHK cells representing an exception in which both viruses produced comparably large syncytia.

Together, these data show that syncytium formation is not a fixed, virus-intrinsic property dictated by F cleavage site sequence alone. Instead, the same virus can display markedly different fusion phenotypes depending on the cellular context. This prompted us to next define how furin-like versus trypsin-like protease environments differ across respiratory models and patient-derived airway compartments.

### Furin-like and trypsin-like proteases predominate in patient airways and *in vitro* models

The pronounced, cell type–dependent differences in syncytium formation observed in Fig. 1 prompted us to ask whether the respiratory protease environment differs across airway compartments and experimental model systems in a way that could account for these phenotypes.

**Fig 2:**
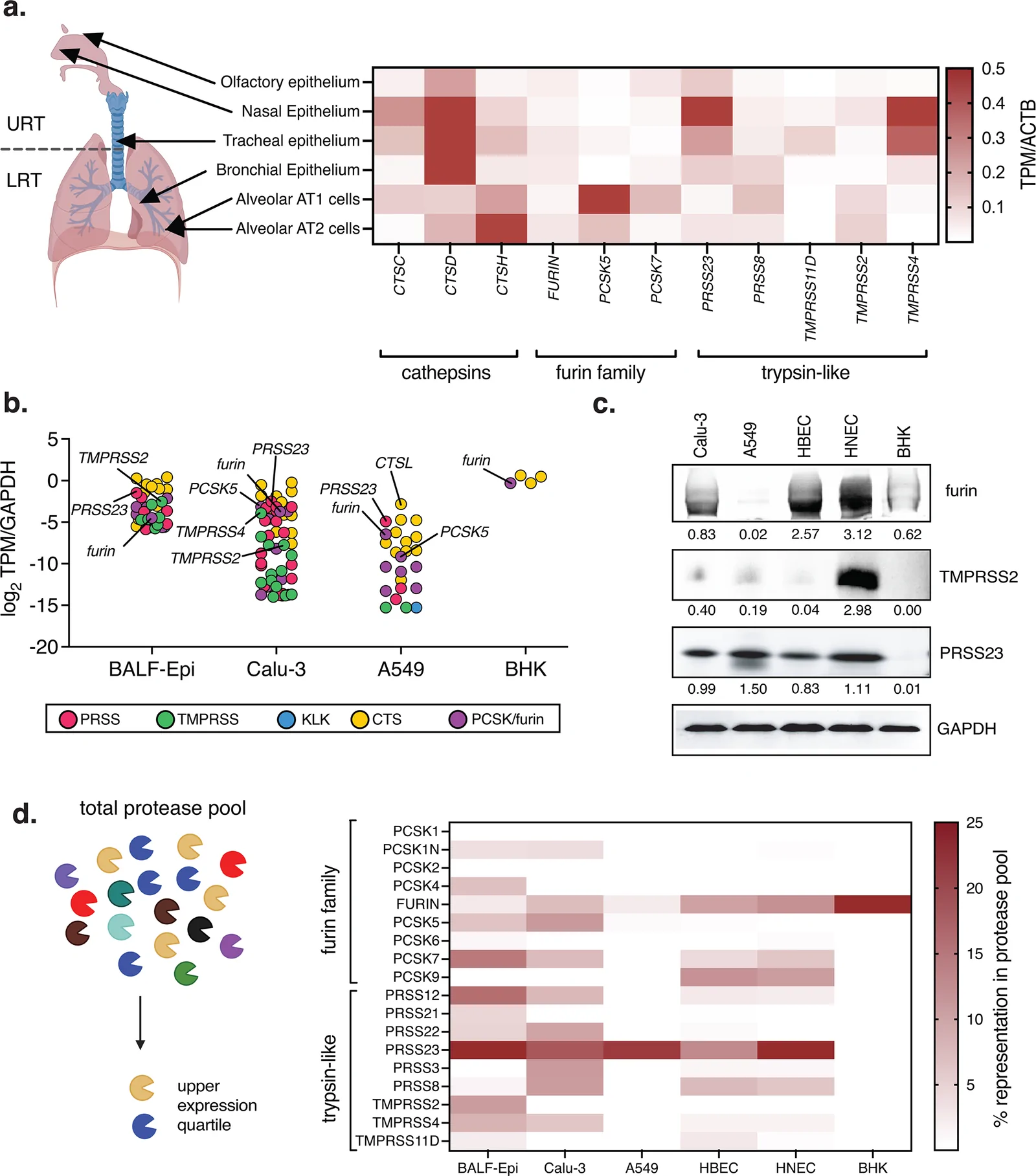
Transcriptomic and protein-based profiling of furin-like and trypsin-like proteases in patient airway compartments and *in vitro* models. (**a**) Bulk transcriptomic analysis of protease expression across epithelial populations from the human upper and lower respiratory tract compiled from publicly available data sets (Table 1). Expression values (TPM) were normalized to ACTB (*n* = 3 samples per compartment). (**b**) Single-cell transcriptomic analysis of protease expression in Calu-3, A549, and BHK cell lines and bronchoalveolar lavage fluid (BALF)-derived epithelial cells from publicly available data sets (Table 1). Expression values (TPM) were normalized to GAPDH (*n* = 3 samples per group). (**c**) Western blot analysis of Calu-3, A549, hBEC, hNEC, and BHK cell lysates. Membranes were probed for furin, PRSS23, TMPRSS2, and GAPDH (loading control) (*n* = 1 per cell type). Relative abundance values of densitometry (band/GAPDH) are represented in numbers underneath each blot. (**d**) Top-quartile analysis of protease expression from panel b. Proteases were ranked by expression within each data set, the top 25% were selected, and the fraction classified as trypsin-like or furin-like is shown for each cell type.

Across publicly available transcriptomic data sets spanning upper and lower airway epithelial populations (19, 20), two protease classes dominated the respiratory proteolytic landscape: furin-family proprotein convertases (PCSKs) and trypsin-like serine proteases (including PRSS and TMPRSS family members) (Fig. 2a). Strikingly, their relative abundance varied by compartment, revealing an inverse gradient along the tract: upper airway epithelial cells were enriched for trypsin-like proteases (including PRSS23 and multiple TMPRSS family members), whereas lower airway and alveolar populations showed comparatively higher expression of furin and related PCSKs (Fig. 2a). Cathepsins (CTSC, CTSD, and CTSH) were also detected across the tract, with broadly uniform CTSD expression and comparatively higher CTSH in lower airway and alveoli (Fig. 2a); however, as neither HPIV3 nor RSV F is known to depend on cathepsin-mediated cleavage, we focused subsequent analyzes on the furin-like and trypsin-like classes.

**TABLE 1:** Data used for transcriptomic analysis of protease expression in Fig. 2

Tissue/cell type	Data accession number	Reference
Nasal epithelium	GSE97668	(21)
Olfactory epithelium	GSE80249	(19)
Tracheal epithelium	GSE101993	(22)
Bronchial epithelium	GSE118761	(20)
Alveolar epithelium AT1 cells	GSE86618	(23)
Alveolar epithelium AT2 cells	GSE166703	(24)
BALF-epithelial cells	GSE145926	(25)
Calu 3 cells	GSE148729	(26)
A549 cells	GSE106261	(27)
BHK-21 cells	GSE124122	(28)
hBEC	GSE61955	(29)
hNEC	PRJNA996592	(30)

To determine how well commonly used experimental systems reflect these *in vivo* patterns, we analyzed protease expression in Calu-3, A549, and BHK cell lines alongside BALF-derived epithelial samples (21; Fig. 2b). These models span distinct protease environments: A549 cells were trypsin-like–dominant (high PRSS23, low furin), BHK cells were furin-dominant (minimal protease expression beyond furin), and Calu-3 cells exhibited a mixed profile with both furin and TMPRSS family members (including TMPRSS2 and TMPRSS4) (Fig. 2b). To allow comparison across genes and samples, we expressed each protease as its transcripts per million (TPM) value divided by that of a stable housekeeping gene (TPM/ACTB in Fig. 2a, TPM/GAPDH in Fig. 2b); the housekeeping gene was selected within each data set as the one with the lowest variance across samples, providing a stable reference. Higher values therefore indicate greater protease expression relative to this reference.

We next asked whether these transcript-based classifications are reflected at the protein level in the same cell types used in Fig. 1 (Fig. 2c). Western blotting supported the same dominant-protease assignments: A549 cells showed low furin and robust PRSS23; Calu-3 cells, primary hBECs, and hNECs showed strong furin expression (with TMPRSS2 additionally detected in hNECs); and BHK cells showed high furin but no detectable PRSS23.

To summarize protease dominance more systematically, we performed a top-quartile expression analysis of the trypsin-like and furin-like protease pool (Fig. 2d). PRSS23 ranked among the most highly expressed proteases in all models except BHK cells, whereas furin was highly represented across all systems except A549 cells, reinforcing a consistent separation between trypsin-like–dominant and furin-dominant environments across *in vitro* and *ex vivo* systems.

Collectively, these analyses show that (i) furin-like (PCSK) and trypsin-like serine proteases dominate the respiratory protease landscape, (ii) their relative abundance varies across upper versus lower airway compartments in patients, and (iii) commonly used *in vitro* models occupy distinct positions along this same axis. This provides a mechanistic framework for the cell type–dependent differences in syncytium formation and spread observed in Fig. 1.

### RSV preferentially relies on furin, whereas HPIV3 uses redundant protease pathways

Given the distinct trypsin-like versus furin-like protease environments identified in Fig. 2, we next asked whether RSV and HPIV3 differ in their intrinsic protease requirements for fusion protein activation. Specifically, we tested the prediction that RSV is preferentially activated by furin-like proteases, whereas HPIV3 can be activated redundantly by both furin-like and trypsin-like proteases; in summary, RSV is expected to show a furin preference, while HPIV3 is expected to retain protease redundancy.

**Fig 3:**
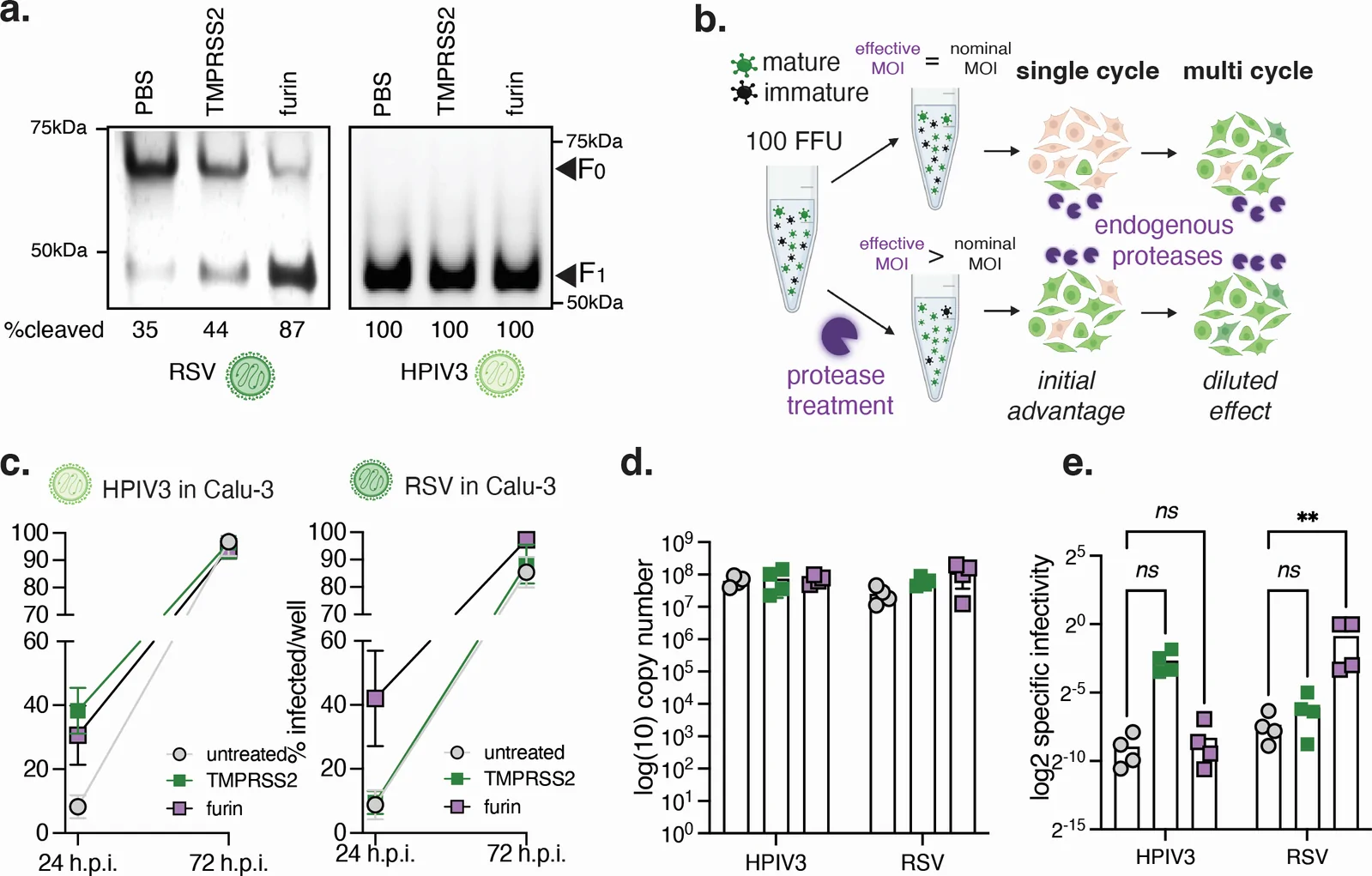
Protease pre-treatment assays to assess fusion protein processing and particle infectivity. (**a**) Western blot analysis of HPIV3 and RSV virions following pre-treatment with recombinant furin (0.1 ng/µL) or recombinant TMPRSS2 (0.1 ng/µL) for 30 min at 37°C. Input virus was normalized to 10,000 FFU/mL (HPIV3) or 56,000 PFU/mL (RSV). F protein processing was detected using anti-HPIV3 F or anti-RSV F antibodies (*n* = 1). The percentage of cleaved F protein (%cleaved), calculated by densitometry as F1 / (F0 + F1), is indicated below each lane. (**b**) Conceptual schematic illustrating how protease-dependent maturation raises the effective multiplicity above the fixed nominal input, adapted to the experimental organization shown throughout this figure. (**c**) Percent infected Calu-3 cells at 24 h and 72 h post-infection following protease pre-treatment of viral stocks and infection at MOI 0.1. Infection was quantified by high-content imaging based on GFP-positive cells (*n* = 3 biological replicates per condition). (**d**) Viral genome copy number in supernatants from (**c**) quantified by RT-qPCR using a GFP standard curve. Data points represent biological replicates from *n* = 4 independent experiments. (**e**) Specific infectivity calculated as infectious units per genome copy number from (**d**). Infectious titers were determined by focus-forming assay on Calu-3 cells. Data points represent biological replicates from *n* = 4 independent experiments; statistical significance was assessed by two-way ANOVA. * *P* < 0.05, ** *P* < 0.01, *** *P* < 0.001, **** *P* < 0.0001, ns = not significant.

To directly test protease-dependent processing of the viral fusion (F) proteins, we treated viral stocks with recombinant furin or TMPRSS2 and assessed F cleavage by Western blot (Fig. 3a). RSV showed a clear furin preference: furin treatment robustly processed RSV F, whereas TMPRSS2 had minimal effect. In contrast, HPIV3 F was efficiently processed by both furin and TMPRSS2, and substantial cleavage was detectable even in untreated stock, consistent with broad protease compatibility under our production conditions.

We next asked how these differences in F processing translate into infection outcomes by evaluating the effects of protease pre-treatment under single- and multi-cycle conditions (Fig. 3b). Infections were performed at low MOI (0.1) to permit subsequent spread, and percent infected cells was quantified at 24 and 72 h post-infection (Fig. 3c). At 24 h post-infection—corresponding to a single replication cycle under these conditions—furin pre-treatment significantly increased the fraction of RSV-infected cells in Calu-3 cultures, whereas TMPRSS2 had little effect. By contrast, HPIV3 infection was enhanced by pre-treatment with either furin or TMPRSS2, consistent with redundant protease usage. However, by 72 h post-infection, infection levels converged across all conditions for both viruses, indicating that the initial advantage of pre-cleaved particles is diluted over multiple rounds of replication as endogenous host proteases support ongoing activation during spread. This time-dependent convergence is consistent with a protease-dependent contribution to the effective multiplicity of infection: pre-treatment with a compatible protease increases the fraction of fusion-competent particles, raising the effective multiplicity above the fixed nominal input (MOI 0.1) at the single-cycle timepoint, while by 72 h, endogenous host proteases equalize activation across conditions, as schematically depicted (Fig. 3b).

To distinguish effects on particle infectivity from effects on particle abundance, we quantified viral genome copy numbers and infectious titers following protease pre-treatment. Genome copy numbers were comparable across conditions for both viruses (Fig. 3d), indicating that total particle output was not substantially altered. In contrast, furin pre-treatment significantly increased RSV-specific infectivity (infectious units per genome copy), whereas HPIV3-specific infectivity was not significantly changed by protease pre-treatment (Fig. 3e). Together, these data indicate that furin-dependent maturation is a key determinant of RSV particle infectivity, while HPIV3 can achieve efficient infectivity through multiple protease pathways.

### TMPRSS2 supplementation alters HPIV3 spread and syncytium formation in a protease-dependent context

Having established that RSV and HPIV3 differ in their intrinsic protease requirements for maturation, we next investigated how changes in protease availability influence viral spread and syncytium formation.

**Fig 4:**
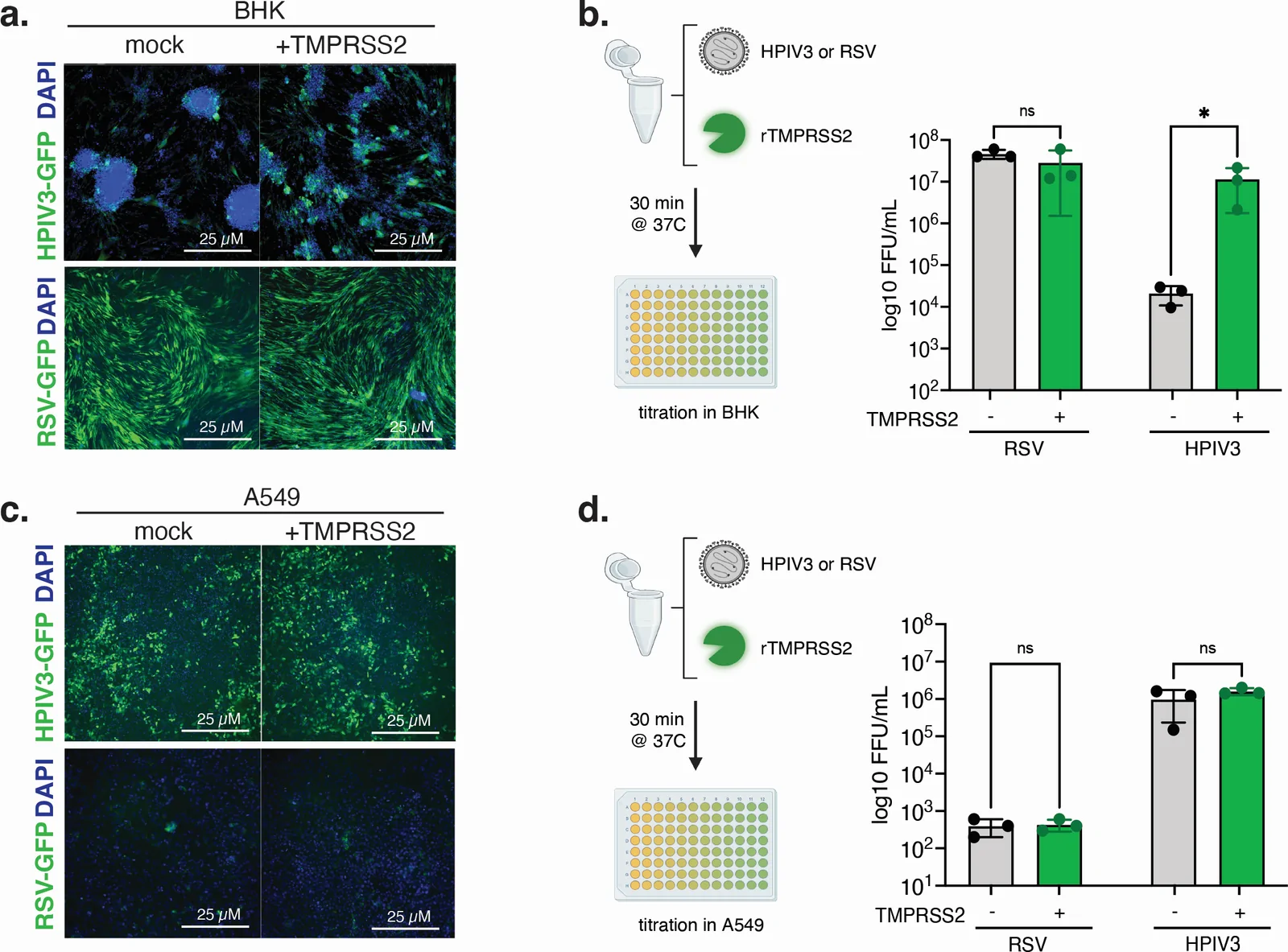
Protease supplementation assays to quantify effects of exogenous TMPRSS2 on viral spread and syncytium formation. (**a**) Representative fluorescence microscopy images of HPIV3- and RSV-infected BHK cells at 48 hpi with or without recombinant TMPRSS2 (10 ng/mL) added at the time of infection. Infection was performed at MOI 0.1 (*n* = 3 biological replicates). (**b**) Infectious titers (focus-forming units per mL) in supernatants from panel a at 48 hpi. Data points represent biological replicates (*n* = 3); statistical significance was assessed by one-way ANOVA. (**c**) Representative fluorescence microscopy images of HPIV3- and RSV-infected A549 cells at 48 hpi with or without recombinant TMPRSS2 (10 ng/mL) added at the time of infection. Infection was performed at MOI 0.1 (*n* = 3 biological replicates). (**d**) Infectious titers (FFU/mL) in supernatants from panel c at 48 hpi. Data points represent biological replicates (*n* = 3). * *P* < 0.05, ns = not significant.

To test this, we supplemented cells with recombinant TMPRSS2 in a system dominated by furin activity. BHK cells express high levels of furin but minimal trypsin-like proteases (Fig. 2b), providing a model to assess the impact of introducing serine protease activity. Under these conditions, both viruses formed large syncytia at baseline (Fig. 4a). Addition of recombinant TMPRSS2 markedly altered HPIV3 spread, resulting in increased dissemination accompanied by a reduction in syncytium formation, quantified as nuclei per infected region of interest (ROI) (Fig, S1). In contrast, RSV spread and syncytium morphology were largely unaffected by TMPRSS2 supplementation (Fig. 4a).

To determine whether this shift reflects increased production of infectious particles, we next quantified viral titers following TMPRSS2 treatment. In BHK cells, TMPRSS2 supplementation significantly increased HPIV3 infectious titers, whereas RSV titers remained unchanged (Fig. 4b). These findings are consistent with a shift in HPIV3 toward cell-free spread in the presence of trypsin-like protease activity.

We next asked whether this effect is dependent on the baseline protease environment by examining A549 cells, which are enriched for trypsin-like proteases and express low levels of furin (Fig. 2). In this context, HPIV3 spread efficiently without forming large syncytia, and supplementation with TMPRSS2 did not further enhance spread (Fig. 4c). RSV infection remained limited and was similarly unaffected by TMPRSS2.

Consistent with these observations, TMPRSS2 supplementation did not significantly alter infectious titers for either virus in A549 cells (Fig. 4d), indicating that endogenous protease activity in this system is sufficient to support HPIV3 spread and insufficient to rescue RSV.

Together, these data demonstrate that protease availability can actively redirect HPIV3 between syncytia-dependent and cell-free modes of spread, whereas RSV remains largely constrained by its dependence on furin-mediated processing.

### Serine protease inhibitors (SERPINs) selectively inhibit viral spread and syncytium formation

**Fig 5:**
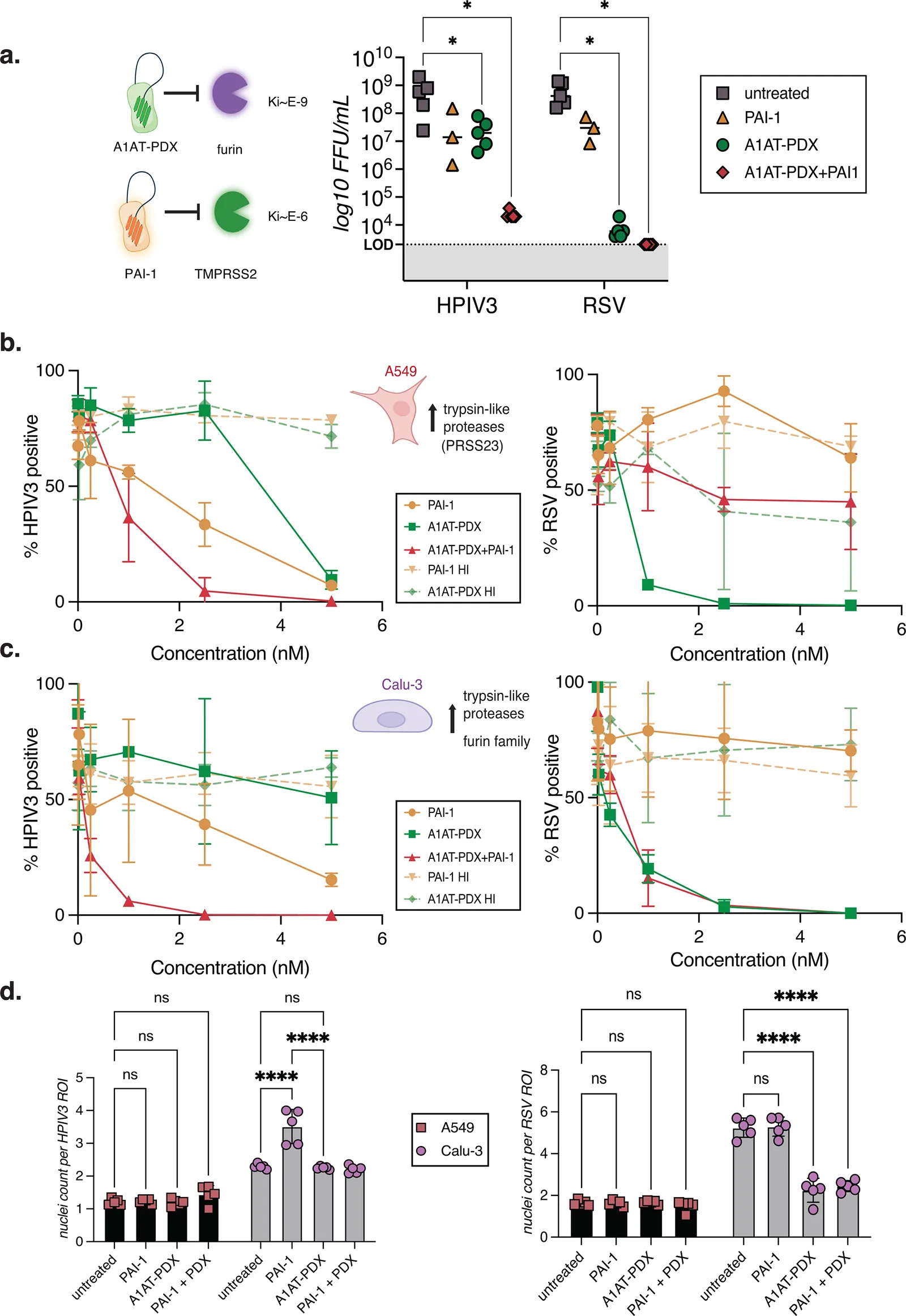
SERPIN inhibition assays to test protease pathway dependence of infection and syncytium architecture. (**a**) Infectious titers at 72 hpi in Calu-3 cells treated with the indicated SERPINs (5 nM) or vehicle control (phosphate-buffered saline). Treatments included PAI-1, A1AT-PDX, or A1AT-PDX + PAI-1; schematic of targeted protease classes is shown (left). Data points represent biological replicates (*n* = 5); statistical significance was assessed by two-way ANOVA. (**b**) Percent infected A549 cells at 48 hpi quantified by high-content fluorescence microscopy following treatment with recombinant PAI-1, recombinant A1AT-PDX, heat-inactivated (HI) controls, or combination treatment across 0–5 nM. Data are mean ± SEM (*n* = 5 biological replicates). (**c**) Percent infected Calu-3 cells quantified as in panel b following the same treatments across 0–5 nM. Data are mean ± SEM (*n* = 5 biological replicates). (**d**) Syncytium size quantified as nuclei per region of interest (ROI) per well in HPIV3- and RSV-infected A549 and Calu-3 cells following treatment with PAI-1, A1AT-PDX, or combination treatment (2.5 nM). Data points represent biological replicates (*n* = 5); statistical significance was assessed by two-way ANOVA. * *P* < 0.05, ** *P* < 0.01, *** *P* < 0.001, **** *P* < 0.0001, ns = not significant.

Protease availability shapes viral spread and can be experimentally manipulated (Fig. 4), raising the question of whether host-derived antiproteases are sufficient to restrict infection in a protease-specific manner. To test this, we treated infected cells with recombinant serine protease inhibitors (SERPINs) targeting distinct protease classes. A1AT-PDX, a potent inhibitor of furin-like proteases (furin and related convertases such as PC5/6), and PAI-1, which inhibits TMPRSS2 (31) and other redundant trypsin-like proteases (e.g., uPA and TMPRSS11D) (32), were used individually or in combination (Fig. 5a). In Calu-3 cells, inhibition of furin activity alone was sufficient to markedly reduce RSV infectious titers, whereas inhibition of trypsin-like proteases had minimal effect. In contrast, attenuation of HPIV3 required combined inhibition of both protease classes, with A1AT-PDX and PAI-1 together producing the strongest reduction in viral titers (Fig. 5a). These findings are consistent with a dominant reliance of RSV on furin-mediated processing and a more flexible protease usage by HPIV3.

We next examined how these effects depend on the underlying protease environment using A549 cells, which are enriched for trypsin-like proteases and express low levels of furin (Fig. 2). Under these conditions, PAI-1 treatment resulted in a strong, dose-dependent reduction in HPIV3 infection, whereas A1AT-PDX had only a modest effect (Fig. 5b). In contrast, RSV infection was selectively inhibited by A1AT-PDX, with little to no effect of PAI-1. These data indicate that SERPIN-mediated inhibition reflects the protease dependencies observed in Fig. 3 within a distinct cellular context.

In protease-rich environments, multiple pathways may support viral spread simultaneously. In Calu-3 cells, which express both furin and TMPRSS family proteases, combined treatment with A1AT-PDX and PAI-1 produced the strongest reduction in infection for both viruses (Fig. 5c), indicating that parallel proteolytic routes contribute to fusion protein activation in this system. Across all treatments and concentrations, inhibitor exposure did not reduce cell viability, confirming that these effects reflect protease inhibition rather than cytotoxicity (Fig, S2).

Finally, we assessed the impact of antiprotease treatment on syncytium formation. In HPIV3-infected Calu-3 cells, inhibition of trypsin-like proteases with PAI-1 resulted in increased syncytium size, consistent with a shift toward furin-dependent fusion under these conditions (Fig. 5d). Conversely, inhibition of furin with A1AT-PDX reduced syncytium size in RSV-infected cells, indicating that furin-mediated processing promotes extensive syncytium formation. These results demonstrate that antiprotease activity not only limits infection but also modulates the mode of viral spread.

Together, these data show that host-derived antiproteases can selectively restrict viral spread by targeting the protease pathways required for fusion protein activation. RSV was preferentially sensitive to furin-pathway inhibition, whereas HPIV3 required broader inhibition across protease classes, consistent with its redundant protease usage.

## DISCUSSION

The mode of viral spread is a central determinant of pathogenesis in respiratory infections, yet it is often treated as a fixed, virus-intrinsic property (33). Here, we show that for two closely related respiratory viruses, RSV and HPIV3, the balance between syncytia-dependent and cell-free spread is not hardwired but instead reflects the local proteolytic environment. By integrating phenotypic analyses, protease landscape profiling, and targeted perturbations, we propose a model in which protease availability dictates fusion protein processing, which in turn governs spread modality and infection dynamics.

At the level of fusion activation, our data support a two-step logic: cleavage-site sequence constrains which proteases *can* activate F, while the local airway milieu determines which proteases are actually *used*. Importantly, protease engagement is not dictated by motif class alone; rather, subtle differences in residue composition and presentation shape how efficiently a protease can access and process the substrate. In this context, RSV exhibited a strong preference for furin-mediated processing, with infectivity and maturation largely insensitive to TMPRSS2 (Fig. 3). In contrast, HPIV3 displayed functional redundancy, with efficient activation supported by both furin-like and trypsin-like proteases. Together, these findings suggest that cleavage-site biochemistry establishes a framework of protease compatibility that is then selectively engaged by the host protease landscape (34–36).

We further show that host protease landscapes vary across both respiratory tract compartments and *in vitro* models. Transcriptomic profiling revealed an inverse gradient of trypsin-like and furin-like proteases, with upper airway environments enriched for serine proteases and lower airway environments exhibiting higher furin expression (Fig. 2). This pattern was recapitulated in widely used cell lines and tracked with cell type–dependent differences in syncytium formation (Fig. 1) and is consistent with other reports in the literature (37). In furin-dominant contexts, both viruses formed larger syncytia, whereas increased trypsin-like protease availability was associated with smaller syncytia and more cell-free spread. Consistent with its broader protease compatibility, HPIV3 could be redirected toward increased cell-free dissemination upon serine protease supplementation (Fig. 4), while RSV was comparatively insensitive to this perturbation. Together, these findings underscore that spread modality reflects the intersection of intrinsic protease compatibility and local protease availability, with furin activity emerging as a key driver of extensive syncytial fusion.

These differences in protease availability were sufficient to shift spread modality. Protease supplementation and inhibition experiments showed that HPIV3 can transition between syncytia-dependent and cell-free modes depending on which protease activities are available, whereas RSV remained comparatively restricted. In furin-rich systems, addition of TMPRSS2 reduced HPIV3 syncytia and increased infectious titers, consistent with enhanced cell-free spread (Fig. 4), while having minimal impact on RSV. Conversely, SERPIN-mediated inhibition demonstrated that RSV spread could be suppressed by furin inhibition alone, whereas HPIV3 required combined blockade of furin-like and trypsin-like proteases (Fig. 5). Together, these results establish that syncytium formation is a tunable phenotype that emerges from the balance of available proteolytic activities.

A less obvious consequence of protease-dependent activation is that the local proteolytic environment can modulate not only the mode of spread but the effective multiplicity of infection. Because only cleavage-activated particles are fusion-competent (9), the fraction of a viral population capable of initiating productive infection is set by protease availability rather than by total particle number. A stock or a tissue rich in a compatible protease therefore presents a higher effective multiplicity than the same number of particles in a protease-poor environment, even when the nominal input is identical—a principle well established for protease-dependent activation of influenza and other enveloped viruses (38–41). This distinction, illustrated in this manuscript (Fig. 3b), likely underlies the divergence we observe between single-cycle and multi-cycle outcomes: the advantage of pre-activated particles is evident early but is equalized once endogenous proteases support activation across conditions. Extending this logic to the airway, the pronounced gradient in furin-like and trypsin-like activity along the respiratory tract (Fig. 2; [37, 42]) implies that the same inoculum could achieve markedly different effective multiplicities in different compartments—potentially shaping where a virus establishes infection, how efficiently it spreads locally, and the tempo of dissemination along the tract. For viruses such as RSV that depend on a specific protease class, this predicts that effective infectivity is concentrated in compartments supplying that activity, whereas a protease-flexible virus such as HPIV3 would maintain more uniform effective multiplicity across the airway.

Host-derived antiproteases add a further layer of regulation over viral dissemination. SERPIN-mediated inhibition recapitulated virus-specific protease dependencies, with RSV selectively sensitive to furin inhibition and HPIV3 requiring broader inhibition across protease classes (Fig. 5). Importantly, the SERPINs used here act on protease families rather than single enzymes: PAI-1 inhibits TMPRSS2 and other redundant trypsin-like proteases (e.g., uPA, TMPRSS11D, and trypsin), and A1AT-PDX inhibits furin and related proprotein convertases such as PC5/6 (32, 43). We therefore interpret these effects at the level of protease class rather than any individual protease, consistent with the functional redundancy among airway proteases that activate viral fusion proteins. These results highlight that spread is governed not only by activating protease expression but also by the local balance between proteases and antiproteases (41, 44–46). In this framework, syncytium formation reflects competing proteolytic forces: conditions favoring furin activity promote extensive cell–cell fusion (Fig. 1 and 5), whereas increased trypsin-like activity or antiprotease pressure can limit syncytial expansion and bias infection toward extracellular routes (Fig. 4 and 5).

These findings suggest possible implications for respiratory virus pathogenesis. RSV’s strong dependence on furin-mediated processing, together with higher furin expression in lower airway data sets (Fig. 2), is consistent with a model in which RSV may be better positioned to support extensive syncytium formation in furin-rich environments, potentially contributing to epithelial disruption and inflammation that accompany severe disease (47–49). In contrast, HPIV3’s broader protease compatibility may support replication across diverse airway compartments without necessarily producing extensive syncytial pathology under all conditions. More broadly, our data support the idea that tissue-specific protease landscapes can influence how respiratory viruses disseminate and the types of cytopathic effects they induce (46, 50).

Our results also highlight that commonly used *in vitro* systems can impose strong biases on observed phenotypes. A549 and BHK cells represent extremes of protease environments that favor cell-free or syncytial spread, respectively (Fig. 1 and 2), and may therefore skew interpretation of “virus-intrinsic” determinants of spread. Accounting for protease context will be important for comparing results across systems and for selecting models that best reflect relevant airway compartments.

From a therapeutic perspective, host proteases have long been recognized as attractive antiviral targets because they are genetically stable and can be shared across multiple respiratory viruses (38, 46, 51). Inhibitors of trypsin-like airway proteases (including TMPRSS family members) and strategies targeting furin-like proprotein convertases have been explored in the context of influenza, coronaviruses, and other enveloped viruses (52). Several such inhibitors have advanced to clinical testing: the TMPRSS2 inhibitors camostat and nafamostat were evaluated in COVID-19 trials, though with limited clinical benefit, and furin-directed strategies have been explored preclinically. These experiences underscore both the therapeutic interest in host proteases and the challenges of translating protease inhibition into clinical efficacy (42, 53, 54). Our results extend this concept by suggesting that host-directed modulation of protease activity may not only reduce infection but also reprogram spread modality. At the same time, selective protease inhibition may have unintended consequences: for viruses with redundant activation pathways, suppressing one protease class (e.g., trypsin-like serine proteases) could bias fusion activation toward alternative routes (e.g., furin-like processing), potentially shifting infection toward more syncytia-dependent spread rather than uniformly suppressing dissemination. Thus, therapeutic approaches that target the protease landscape will likely require careful matching to virus-specific dependencies and consideration of pathway compensation in the airway protease–antiprotease network.

Several limitations and future directions follow from this work. First, while our data link protease environments to spread modality in cell culture, testing this model in more physiologically complex systems—such as differentiated airway epithelium, *ex vivo* tissue, and *in vivo* infection models—will be important to establish how protease-dependent switching operates within the intact airway. Second, our protease “landscape” measurements are largely transcript- and abundance-based; however, protease function is ultimately determined by activity, which can be shaped by zymogen activation, trafficking, localization (secretory pathway versus cell surface), post-translational modifications, and endogenous inhibitors (55–57). Defining where and when furin-like versus trypsin-like activities are available during infection (and whether they colocalize with F at virions or the infected-cell membrane) will therefore be critical. Third, the airway proteolytic network is broader than the markers examined here, and additional proteases, antiproteases, and spatial constraints (e.g., compartment-specific expression, mucus-associated inhibitors, or inflammation-driven remodeling) may further tune spread. Finally, although our results support distinct protease preferences, the structural determinants that govern enzyme–substrate engagement are likely multifactorial and may depend on conformational dynamics and glycosylation of both proteases and fusion proteins (58); resolving these determinants will require dedicated structure–function and biochemical studies.

In summary, our data support a conceptual shift in which viral spread modality is not a fixed trait but a tunable outcome dictated by the host proteolytic landscape. Across respiratory models, the balance between furin-like and trypsin-like protease activities shaped whether infection proceeded predominantly through syncytium formation or extracellular virion production, with RSV exhibiting a strong preference for furin-mediated processing and HPIV3 retaining functional redundancy across protease classes. Host-derived antiproteases further modulated this balance, underscoring that syncytium formation is a controllable phenotype emerging from competing proteolytic activities. Together, this framework links protease availability to fusion activation and dissemination strategy, highlights how *in vitro* systems can bias inferred virus behavior, and suggests that host-directed modulation of protease pathways may offer a route to control respiratory virus infection.

## MATERIALS AND METHODS

### Cells and viruses

A549 (ATCC, CCL-185), Calu-3 (ATCC, HTB-55), and BHK-21 (ATCC, CCL-10) cells were maintained in Dulbecco’s Modified Eagle Medium (Corning, 10-013-CV) supplemented with 10% fetal bovine serum and 1% penicillin–streptomycin at 37°C in a humidified incubator with 5% CO₂. Primary human bronchial epithelial cells (hBECs) BCi NS1.1 cells (obtained from the laboratories of Drs. Matthew Walters and Ronald Crystal, Cornell University) and human nasal epithelial cells (hNECs) (obtained as a gift from the laboratory of Dr. Andrew Pekosz) were cultured in PneumaCult-Ex Plus Medium (STEMCELL Technologies, #05040) in monolayer.

Recombinant HPIV3-GFP strain JS was a gift from Dr. Peter Collins. RSV-GFP strain A2 was a gift from Dr. Charles Rice. Viral stocks were propagated in LLC-MK2 cells and concentrated, and infectious titers were determined using assay-specific readouts: HPIV3-GFP was titrated by focus-forming assay on Calu-3 cells and reported as focus-forming units per mL (FFU/mL), whereas RSV was titrated by plaque assay and reported as plaque-forming units per mL (PFU/mL). All infections were performed at the indicated multiplicity of infection (MOI).

### Protease treatment and inhibition

For protease pre-treatment experiments, viral stocks were incubated with recombinant human furin (PreProTech # 450-47-100UG) or recombinant TMPRSS2 (Biomatik, RPC24995) from 10 ng/µL to reach a final treatment concentration of 0.1 ng/µL. Incubation took place for 30 min at 37°C prior to infection.

For protease supplementation experiments, recombinant TMPRSS2 (Biomatik, # RPC24995) was added directly to cell culture media at a final concentration of 10 ng/mL at the time of infection.

For inhibition studies, cells were treated with recombinant active human PAI-1 (Abcam; N150K, K154T, Q319L, M354I mutant) and/or A1AT-PDX (Thermo Scientific # RP070) at the indicated concentration range (0–5 nM). The inhibitory activity of both recombinant PAI-1 and A1AT-PDX was verified by the manufacturers’ certificates of analysis; reagents were used within their certified activity specifications. Where indicated, heat-inactivated controls were generated by incubating proteins at 96°C for 15 min followed by 15 min of incubation at room temperature. Treatments were added at the time of infection and maintained throughout the experiment.

### Multi-cycle infection assays

Cells were infected at a multiplicity of infection (MOI) of 0.1 to allow multi-cycle replication. Following adsorption for 90 min at 37°C, inoculum was removed and replaced with fresh media containing the indicated treatments. Infection was allowed to proceed for 24 h (single-cycle conditions) or 72 h (multi-cycle conditions), after which cells or supernatants were harvested for downstream analysis.

### High-content imaging and syncytium quantification

Infected cells were fixed at the indicated time points using 4% paraformaldehyde and stained with DAPI to visualize nuclei. Infection was monitored via GFP reporter expression encoded by HPIV3.

High-content imaging was performed using a Cellomics CX7 high-content screening microscope (Thermo Fisher Scientific, HCSDCX7LEDPRO). Regions of infection were defined based on GFP-positive signal, and nuclei were identified via DAPI staining using the Colony Formation BioApplication in Cellomics HCS Studio (CellInsight). This makes it reproducible and names the standard app you confirmed.

Syncytia were quantified as the number of nuclei per GFP-positive region of interest (ROI). Data represent the mean number of GFP-positive ROIs in 36 fields per well and *n* = 5 biological replicates.

### Focus-forming assay

Viral titers were determined by focus-forming assay (FFA) on Calu-3 cells for GFP-reporter viruses (including HPIV3-GFP) and for experiments in which infectious output was quantified by GFP-positive foci. Briefly, cells in 96-well plates were infected with 50 µL of serially diluted supernatants and incubated for 48 h. Cells were then fixed, nuclei were stained with DAPI, and foci were detected by GFP signal. Wells were infected in technical triplicates, and titers are reported as focus-forming units per mL (FFU/mL) using the following formula: FFU/ml = no. of foci/dilution factor × volume of virus (mL). FFU and PFU values reflect assay-specific infectious units and were used as reported for each virus based on the titration method described above.

### Quantification of viral genome copy number

Viral RNA was extracted from supernatants using the QIAamp Viral RNA Mini Kit (Qiagen, 52904) according to the manufacturer’s instructions. Genome copy numbers were quantified by reverse transcription-quantitative PCR (RT-qPCR) using GFP-specific primers (forward 5′-ATGGTGAGCAAGGGCGAGGAG-3′; reverse 5′-TTACTTGTACAGCTCGTCCATGCC-3′).

A standard curve was generated using serial 10-fold dilutions of the pSCRPSY-empty-GFP plasmid. Plasmid DNA was maintained at 200 ng/μL, and 8 μL was used as input for the initial dilution series. Based on an estimated plasmid size of ~10 kb, the molar mass was approximated at ~6.6 × 10⁶ g/mol, and genome copy numbers were calculated accordingly.

RT-qPCRs were performed using TaqMan Fast Virus 1-Step Master Mix (Thermo Fisher Scientific, A25741) in MicroAmp Optical 96-well reaction plates (Thermo Fisher Scientific, N8010560) on a QuantStudio 5 Real-Time PCR System (Thermo Fisher Scientific, A34322). Data are expressed as genome copies per mL based on standard curve interpolation.

### Western blot and SDS-PAGE analysis

Cells were lysed in 50 mM Tris-HCl (pH 7.4–8.0), 150 mM NaCl, 1% NP-40 (or Triton X-100), 0.5%–1% sodium deoxycholate, and 0.1% SDS supplemented with cOmplete protease inhibitor (Tablets, Roche, 04693116001). Protein samples were mixed with NuPAGE LDS sample buffer (4×; Thermo Fisher Scientific, B0007) and reducing agent (10×; Thermo Fisher Scientific, B0009), boiled at 95°C for 5 min, and resolved on 4%–12% Bis-Tris gels (Thermo Fisher Scientific, NP0322BOX).

Proteins were transferred to PVDF membranes using the iBlot 2 Dry Blotting System (Thermo Fisher Scientific, IB21001). Membranes were blocked in 5% milk in TBS-T and probed with primary antibodies against furin, PRSS23, TMPRSS2, and GAPDH (Thermo Fisher Scientific, MA5-15738-HRP), followed by HRP-conjugated secondary antibodies (Invitrogen, G21040 and G21234). Signals were detected using HRP Substrate (Thermo Scientific 34076) and imaged using ChemiDoc Imaging System (Bio-Rad 12003153). Membranes were auto-cropped for analysis on the ChemiDoc instrument and further cropped to size for figure panels on Adobe Illustrator. Densitometric quantification of F protein cleavage was performed in ImageJ/Fiji (NIH). For each lane, F0 and F1 band intensities were measured from background-subtracted images, and the percentage of cleaved F protein (%cleaved) was calculated as F1/(F0 + F1) × 100. Values reported in Fig. 3a are from a single experiment (*n* = 1). For Fig. 2c, band intensities for furin, TMPRSS2, and PRSS23 were quantified in ImageJ/Fiji and normalized to the GAPDH loading control in the same lane; normalized values (representative blot, *n* = 1) are shown below each lane.

Purified RSV stocks were used for F protein cleavage analysis (Fig. 3). F cleavage was analyzed by SDS-PAGE followed by silver stain (Pierce Silver Stain Kit, ThermoFisher #24612) and imaged using the ChemiDoc imaging system (Bio-Rad 12003153). Densitometric quantification of F protein relative cleavage was performed as described above.

### Transcriptomic analysis

Publicly available transcriptomic data sets from human respiratory tract epithelial populations and cell lines were analyzed with detailed sources available in Table 1. Data files containing expression values were obtained as transcripts per million (TPM) and normalized to housekeeping genes selected based on minimal variance across samples using R software (R 4.5.3 2026). ACTB and GAPDH were used for normalization in Fig. 2a and b, respectively.

Proteases were classified as trypsin-like or furin-like based on gene family, namely, PRSS and TMPRSS families for trypsin-like proteases and PCSK family for furin-like proteases. For top-quartile analysis, proteases were ranked by expression level within each data set, and the top 25% were selected for downstream classification.

### Statistical analysis

All statistical analyses were performed using GraphPad Prism v9 (GraphPad Software). Data are presented as mean ± SEM unless otherwise indicated. Statistical significance was assessed using two-way analysis of variance (ANOVA) or one-way ANOVA with Dunnett’s test, as specified in figure legends. A *P*-value < 0.05 was considered statistically significant.

## Data Availability

This study analyzed publicly available data sets; their sources and accession numbers are listed in Table 1. Each data set can be accessed through its respective repository via the accession numbers provided. No new sequencing data sets were generated in this study.

## SUPPLEMENTAL MATERIAL

Supplemental material jvi.00727-26-s0001.docx Figures S1 and S2.

ASM does not own the copyrights to Supplemental Material that may be linked to, or accessed through, an article. The authors have granted ASM a non-exclusive, world-wide license to publish the Supplemental Material files. Please contact the corresponding author directly for reuse.
