# Supplementary material for: Airway protease availability governs a reversible shift between syncytial and cell-free spread
Source: J Virol. 2026 Sep 14;100(10):e00727-26. doi: 10.1128/jvi.00727-26 (PMC13712803; doi:10.1128/jvi.00727-26)
Supplement: Supplementary file 1 [file jvi.00727-26-s0001.docx]

**Supplementary Materials.**

**Supplementary Figure S1. TMPRSS2 reduces HPIV3 but not RSV syncytia formation in BHK cells.**
(A) Representative high-content images of BHK cells infected with HPIV3, untreated or treated with recombinant TMPRSS2, shown as merge and individual channels (furin, HPIV3-GFP, DAPI). The DAPI channel resolves nuclear clustering within syncytia independently of the GFP signal. Scale bars, 50 µm. (B, C) Syncytia quantified as mean nuclei per GFP-positive region of interest (ROI) per well, using the high-content pipeline described in Methods, for (B) HPIV3-GFP and (C) RSV-GFP in mock- versus TMPRSS2-treated BHK cells. Each point represents one independent well (mean nuclei/ROI across all ROIs in that well); horizontal bars indicate the group mean. n = 10 wells per condition. (**** p < 0.0001; n.s.; unpaired t-test).

**Supplementary Figure S2. Protease inhibitor treatments do not affect cell viability.**

Total nuclei per well in A549 and Calu-3 cells treated with PAI-1, A1AT-PDX, A1AT-PDX + PAI-1, or the corresponding heat-inactivated (HI) controls across a concentration range of 0–5 nM, quantified by high-content imaging as described in Methods. Cell numbers were comparable across all treatments and concentrations, indicating no measurable cytotoxicity over the range used in Figure 5. Data are mean ± SD; n = 5 biological replicates.
